# Splenic Myelolipomas in the Domestic Cat—To Operate or Not to Operate?

**DOI:** 10.3390/ani14111700

**Published:** 2024-06-05

**Authors:** Diana J. Kennedy, Helena M. T. Ferreira, Melanie J. Dobromylskyj, Stefan Hobi, Angel Almendros, Paweł M. Bęczkowski

**Affiliations:** 1 Beaumont Veterinary Hospital, 172 Oxford Rd., Kidlington OX5 1EA, UK; dianakennedyvet@live.com; 2 Zoetis Virtual Laboratory, Leatherhead KT22 7LP, UK; helena.tunaferreira@zoetis.com; 3 Finn Pathologists, Histopathology Department, One Eyed Lane, Diss IP21 5TT, UK; melanie.dobromylskyj@finnpathologists.com; 4 Department of Veterinary Clinical Sciences, Jockey Club College of Veterinary Medicine and Life Sciences, City University of Hong Kong, Kowloon Tong, Hong Kong SAR, China; stefhobi@cityu.edu.hk (S.H.); aalmendr@cityu.edu.hk (A.A.)

**Keywords:** myelolipoma, spleen, splenectomy, mass, hyporexia, weight loss, cat, feline

## Abstract

**Simple Summary:**

Myelolipoma of the spleen is a benign tumour rarely described in cats. These masses typically do not spread to other organs, but their growth can push or displace surrounding tissues and lead to pain or discomfort. In this report we describe a cat that presented to the veterinarian with a history of poor appetite and weight loss. Following thorough diagnostic investigations, presenting complaints were attributed to the intra-abdominal growth of a splenic mass. The mass was removed surgically, but the patient died in the recovery period. This case report highlights the need for careful assessment of a risk–benefit ratio when approaching this rare form of neoplasm in cats.

**Abstract:**

Myelolipoma is a benign, typically inert neoplasm of uncertain aetiology that is rarely reported in cats. Although commonly asymptomatic, in some cases, myelolipomas can cause abdominal discomfort or present with rupture and haemorrhage. Here, we describe a rare case of a splenic myelolipoma in a Domestic Long Hair cat in which, after extensive diagnostic investigations, clinical signs of hyporexia and weight loss were attributable to the presence of an intra-abdominal mass. The patient was treated by splenectomy and unexpectedly died during the post-operative period. Although splenectomy appears to be a sensible intervention in symptomatic patients, the optimal management of splenic myelolipomas in cats remains unknown. The risk–benefit ratio of surgical management needs to be carefully considered, and therapeutic intervention should be tailored individually to each patient.

## 1. Introduction

Myelolipomas are benign, non-functional neoplasms that contain adipose tissue and dispersed hematopoietic elements resembling bone marrow [1]. They have been infrequently described in humans and several animal species, including dogs and domestic cats [2,3]. 

The etiopathogenesis of myelolipomas is uncertain as it is not clear whether they represent true neoplasms or rather metaplasia (reversible transformation of one differentiated cell type into another due to chronic hormonal stimulation, or chronic inflammatory responses as a result of injuries that involve necrosis, stress, or infection), hamartomas (benign-appearing masses composed of cells indigenous to the particular site resulting from aberrant differentiation) or choristomas (normal tissue, for example hematopoietic stem cells, misplaced during embryogenesis) [4,5]. 

The adrenal gland is a predominant location for human myelolipoma, with a handful of reports describing extra-adrenal locations, including the thorax, pelvis and retroperitoneal space [6,7,8,9]. Whilst adrenal myelolipomas have not been described in cats, there are occasional reports of hepatic myelolipoma and only six reports of splenic myelolipoma in this species [3,10].

Myelolipomas are typically small in size and discovered incidentally on advanced imaging or at post-mortem examination [3,11]. Whilst mostly an incidental finding, in some cases, large myelolipomas can cause compression on surrounding structures and lead to pain, abdominal discomfort and associated clinical signs of hyporexia and weight loss [12]. Furthermore, there is a risk that some necrotic lesions may rupture and lead to intra-abdominal haemorrhage and shock [6,13,14,15]. 

Given the paucity of information about feline myelolipomas, the most appropriate clinical management of this neoplasm in cats is unknown. Extrapolating data from human medicine, conservative watchful surveillance may be appropriate for asymptomatic cats, whilst surgical intervention should be considered in cats where the presence of myelolipoma is associated with abdominal discomfort or in cases of an imminent risk of rupture and hemoabdomen [16].

In this report, we describe a rare case of splenic myelolipoma, where clinical signs of hyporexia and weight loss were attributed to perceived abdominal discomfort caused by the splenic mass. 

## 2. Case Description

### 2.1. History, Clinical Findings and Diagnostic Investigations

A 13-year-old, 3.2 kg (7.05 lb) female neutered, indoor-only, Domestic Long Hair cat presented with progressive hyporexia and insidious weight loss of several months’ duration. Clinical examination revealed odontoclastic resorptive lesions and a reduced body condition score (BCS) of 3/9. Routine haematology and biochemistry revealed only mildly increased activity of ALT (141 U/L; reference interval (RI): 18–84 U/L). Abdominal ultrasonography disclosed splenomegaly with multifocal to coalescing, hyperechoic, well-defined nodules, which distorted the capsular margin. Ultrasound-guided fine needle aspirates of the spleen performed in general practice were consistent with extramedullary haematopoiesis. Remedial dental treatment of all abnormal teeth resulted in a modest improvement of appetite, but the cat failed to regain weight back to its normal range. 

**Figure 1:**
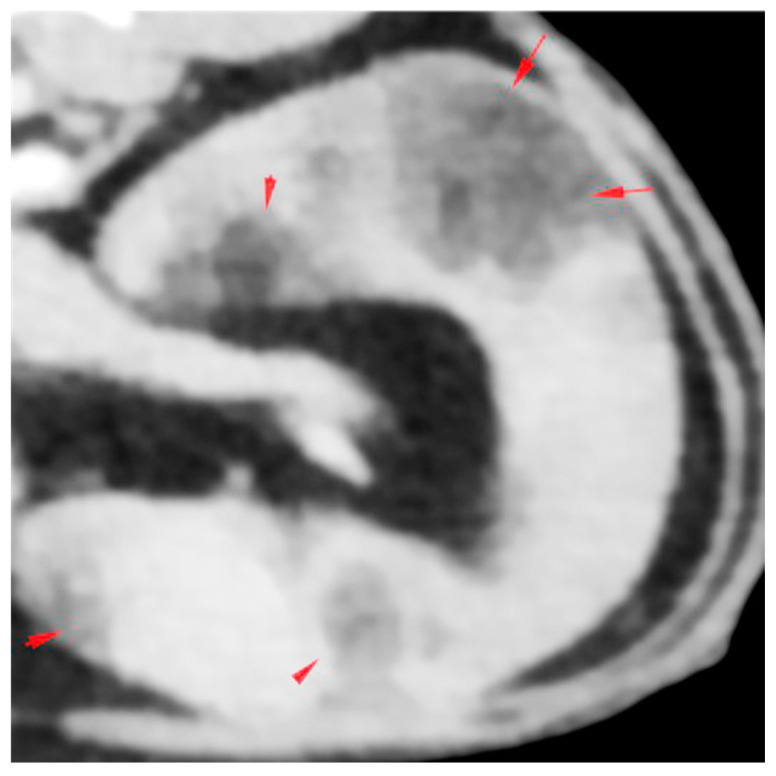
Computed tomography (CT) image of the spleen. The spleen is moderately to severely enlarged, and there are multiple fat-attenuating mass-like lesions within the parenchyma with very mild post-contrast enhancement and a diameter up to 20 mm (red arrows), likely representing myelolipoma.

A referral was prompted for further investigations. A standard small animal database yielded unremarkable haematology results, mildly to moderately increased alanine transaminase (ALT) activity (152 U/L; RI: 18–77 U/L), mildly increased activity of creatinine kinase (CK) (568 U/L; RI: 0–360 U/L), mild hyperglycaemia (9.8 mmol/L; RI: 3.8–7.6 mmol/L) and borderline hypotriglyceridaemia (0.19 mmol/L; RI: 0.20–1.30 mmol/L). Urine was adequately concentrated (urine specific gravity of 1.036) with no evidence of proteinuria or active sediment. Free calcium was increased at 1.36 mmol/L (RI: 1.20–1.32 mmol/L, i-STAT 1 Analyser, Abbot, Princeton, NJ, USA) and parathyroid hormone (PTH) and parathyroid hormone-related peptide (PTH-RP) were lower than 10.0 pg/mL (RI: <40 pg/mL) and 0.1 pmol/L (RI: <0.5 pmol/L), respectively. Serology was negative for Feline Immunodeficiency Virus (FIV) antibodies and Feline Leukaemia Virus (FeLV) antigen, and a low titre of antibodies (1:160) against Feline Coronavirus (FCoV) was detected. Total thyroxine (T4) was within its reference interval (46.6 nmol/L; RI: 7.5–55 nmol/L). A pre-anaesthetic echocardiogram identified a normal left atrium-to-aortic ratio. There were no other abnormalities detected on the echocardiogram. Abdominal ultrasonography showed an enlarged and grossly abnormal spleen with large, diffuse, hyperechoic regions, which distorted the capsular margin and resembled myelolipomas. There also was a bilateral dilation of the renal pelvis and proximal ureters. A small, non-obstructive ureterolith was identified in the left ureter. Subsequent CT evaluation confirmed moderate to severe splenomegaly with multiple, up to 20 mm in diameter, fat-attenuating mass-like lesions within the parenchyma with very mild post-contrast enhancement (Figure 1). Additional CT findings included pyelectasis with distended proximal ureters, mildly distended extrahepatic bile duct (likely age-related) and a single mildly enlarged colonic lymph node. No significant abnormalities were noted on the CT of the thorax.

**Figure 2:**
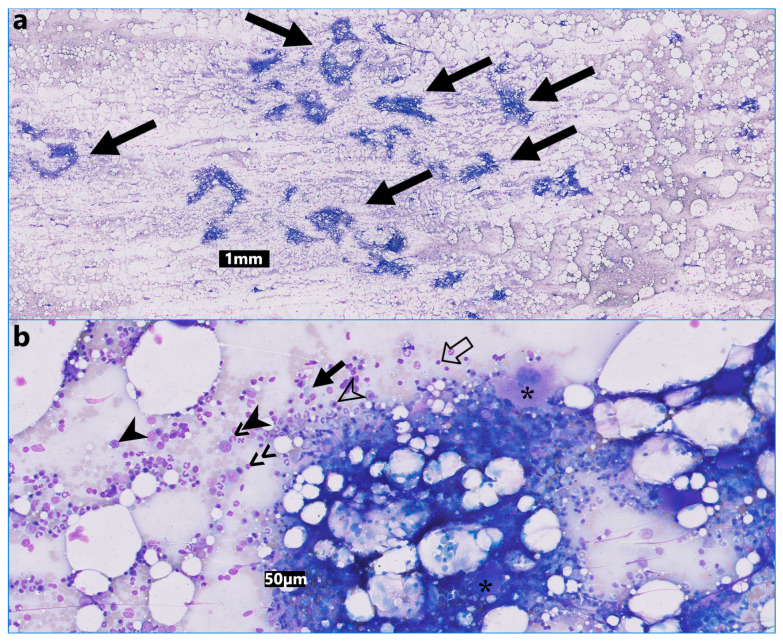
(**a**) Low power of a fine needle aspirate from the splenic masses. Moderate to high nucleated cellularity on a palely basophilic background with large numbers of variably sized lipid globules. The nucleated cells sometimes form aggregates which resemble bone marrow spicules (arrows, modified Wright’s stain). (**b**) High power of a fine needle aspirate from the splenic masses depicting early and late, erythroid and myeloid precursors, megakaryocytes, plasma cells and small lymphocytes (modified Wright’s stain). Megakaryocytes (asterisks), small lymphocyte (open arrow), plasma cell (arrow), early erythroid precursor (full arrowhead), late erythroid precursor (open arrowhead), early myeloid precursor (>>) and late myeloid precursor (arrowhead plus >).

Cytologic examination of aspirates from the fat-attenuating splenic lesions revealed moderate to high nucleated cellularity on a palely basophilic background containing large numbers of variably sized lipid globules (average 56 nucleated cells: 44 fat ratio; range 40:60 to 70:30) and red blood cells (Figure 2). Cellular preservation was moderate, and the nucleated cells sometimes formed aggregates resembling spicules of bone marrow. A 1500-nucleated cell differential count (excluding adipocytes) consisted of 10% early erythroid precursors, 30% late erythroid precursors, 20% early myeloid precursors and 35% late myeloid precursors. Five percent of the nucleated cells comprised mostly small lymphocytes and plasma cells with occasional medium-sized lymphocytes, all within normal morphologic limits. Megakaryocytes were occasionally noted, and some of these cells had a single nucleus or abundant basophilic cytoplasm and concurrent multinucleation. Aspiration from ultrasonographically normal areas of the spleen revealed mild extramedullary haematopoiesis involving the erythrocytic and megakaryocytic cell lineages. Free calcium was repeated twice thereafter, and it was within its reference interval (1.3 mmol/L on both occasions; RI: 1–4 mmol/L). A preoperative manual packed cell volume (PCV) was 32% (RI: 27–50%). No abnormalities were noted on the blood film examination. 

### 2.2. Surgical Case Management and Outcome

**Figure 3:**
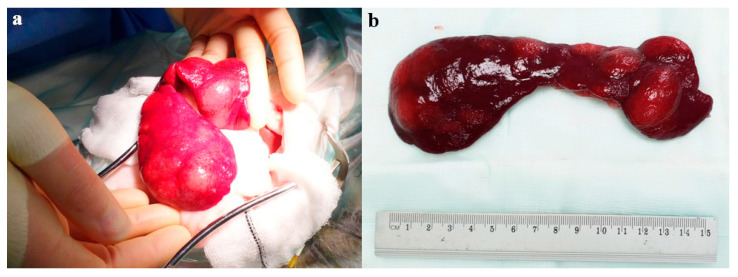
Intra-operative (**a**) and post-operative, pre-fixation (**b**) gross appearance of the irregular-shaped spleen, demonstrating multifocal, variably sized to sometimes locally coalescing, partly raised, moderately well-demarcated and pale, red to tan nodular areas within the splenic parenchyma.

After careful evaluation, the cat was induced with methadone 0.3 mg/kg [0.14 mg/lb] IM, medetomidine 0.04 mg/kg [0.018 mg/lb] IM and ketamine 5 mg/kg [2.27 mg/lb] IM, and underwent elective splenectomy. The spleen was identified with no adherence to any other structures (Figure 3a). Surgery was completed within 20 min.

**Figure 4:**
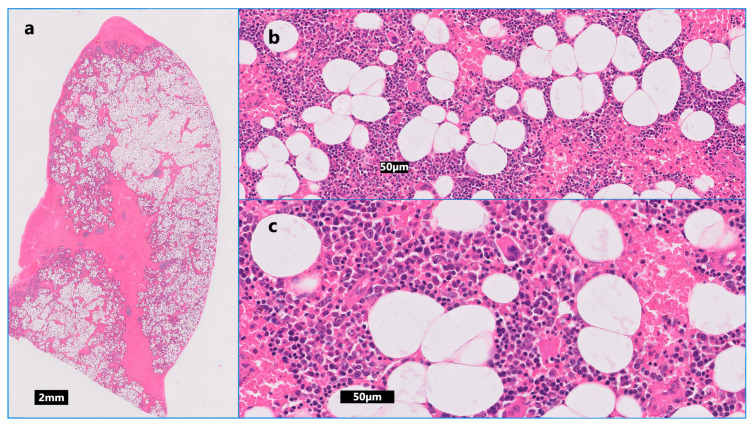
(**a**) Low-power view demonstrating the splenic parenchyma is occupied and expanded by a reasonably well demarcated, expansile, unencapsulated and moderately cellular mass. (**b**,**c**) High-power views of the mass, which is composed of a mixture of variably sized vacuolated cells resembling adipocytes, together with haematopoietic tissues including cells resembling immature and mature myeloid, erythroid and megakaryocytes (haematoxylin and eosin stain).

Grossly, the spleen was moderately enlarged with multifocal, coalescing nodular expansion varying from 1.5 to 3 cm in diameter, deforming the splenic outline and raising the capsule (Figure 3b). No other gross abnormalities were detected at laparotomy, and the spleen was submitted for histopathology. On the cut section, the nodules were moderately well demarcated, multi-lobulated, pale cream/tan in colour, and blended with the adjacent dark splenic parenchyma. Representative sections of the splenic masses and parts of the adjacent normal parenchyma were examined microscopically. The masses were multiple. Low numbers of blast cells contained mitotic figures (low numbers interpreted as normal mitotic activity within this cell type). The histologic morphologic diagnosis was consistent with multifocal splenic myelolipomas (Figure 4). 

The patient initially recovered well from anaesthesia but, after regaining consciousness, demonstrated significant ptyalism and was treated with maropitant (1 mg/kg [0.45 mg/lb], SC, single administration). Approximately three hours after recovery, the patient became hypotensive (45 mmHg; systolic, Doppler RI: 120–140 mmHg) and hypothermic (34.7 °C; normal: 38.1–39.2 °C). Manual post-operative PCV was mildly reduced at 30%. An abdominal scan did not reveal any evidence of post-operative haemorrhage. Blood glucose was moderately increased (16.7 mmol/L; RI: 3.8–8.2 mmol/L) and there was moderate hyponatremia (131 mmol/L; RI: 140–157 mmol/L), moderate to severe hypokalaemia (2.4 mmol/L; RI: 3.4–5.6 mmol/L) and moderate hypochloraemia (97 mmol/L; RI: 111–129 mmol/L). An electrocardiogram (ECG) revealed a sinus rhythm with occasional bigeminal ventricular premature complexes. No significant abnormalities were detected on thoracic radiographs. The patient received intravenous fluid boluses to improve blood pressure (which increased to 122 mmHg systolic), active warming, and subsequent intravenous fluid therapy with 0.9% sodium chloride, supplemented with 30 mmol/L of potassium chloride. The body temperature increased to 37.4 °C, and the ECG showed a normal sinus rhythm. The patient was hospitalised overnight but deteriorated and died in the early hours of the following morning. The forelimbs appeared subjectively colder than the hindlimbs, and a thromboembolic event was speculated to have contributed to sudden death. The coagulation profile testing was not available. A necropsy was not performed, as per the owner’s request.

## 3. Discussion

Myelolipomas are rare neoplasms that occur in various animal species and humans and are frequently diagnosed incidentally [17,18,19,20,21]. Myelolipomas in cats are reported to be either asymptomatic [10] or associated with clinical signs such as hyporexia, vomiting, diarrhoea, depression, anorexia and constipation [2,3,20,22,23]. In the case presented herein, the main clinical sign was hyporexia. As no other underlying causes of hyporexia have been reliably identified during extensive diagnostic investigations, it was presumed that the mass effect was attributed to an enlarged spleen, leading to reduced appetite and weight loss. The decision was made to remove the spleen surgically with curative intent. 

In humans, myelolipomas are also rare and, most commonly, are incidental findings during imaging or autopsy [7,11,24]. The adrenal gland is primarily affected, with rare reports of extra-adrenal locations [7,25]. Myelolipomas tend to present without symptoms unless they haemorrhage following rupture or attain a large size. In the latter cases, surgical excision is recommended [25]. Myelolipomas in cats have been reported in the liver [10,20,22,23], usually associated with peritoneopericardial diaphragmatic hernias [20,23], but are rarely reported in the spleen and have not been described in the adrenal gland. 

The myelolipoma presented in this report occurred in a female cat, and previous reports of myelolipomas in domestic cats included eight females [2,3,4,10,14,20,23,26] and one male [22], suggestive of a female predisposition in this species. In terms of breed predisposition, six feline cases of myelolipoma affected Domestic Short and/or Long Hairs [2,3,10,15,22,23] and there are single reports in a Persian [26] and a Persian cross [20]. The age of the affected cats ranged from 11 to 16 years old, and the myelolipomas were found in the liver or spleen, either as solitary [3,20,26] or multiple lesions [2,22,23] in the same organ. Splenic myelolipomas have also been reported in cheetah [17] and in dogs [18,21]. 

Various clinicopathologic abnormalities have been reported in cats with splenic myelolipomas. Hemoabdomen secondary to rupture of a myelolipoma was reported in one cat with a splenic myelolipoma [2], as well as in a cat with hepatic myelolipoma [15]. One report described progressive weight loss, tenesmus and elevated liver enzymes [2]. Another report described respiratory, gastrointestinal and urinary signs and vocalisation, whilst bloodwork revealed respiratory acidosis with metabolic compensation [2]. In another case with weight loss as the main clinical feature, the cat had a giant splenic myelolipoma and a concurrent hyperthyroidism [27]. Overall, the findings in published reports are non-specific for both splenic and hepatic myelolipomas in cats. Accordingly, even though the majority of known causes of hyporexia and weight loss were excluded in our case, it is difficult to attribute the clinical signs solely to the presence of splenic myelolipoma, as other undetected aetiologies may have contributed to the development of reduced appetite and weight loss. 

The diagnosis of myelolipoma is based on a combination of ultrasonographic [28,29], cytologic and histopathological findings [30,31]. Differential diagnoses for myelolipoma include extramedullary haematopoiesis (EMH), osseous metaplasia, angiomyelolipoma and angiomyomyelolipoma, lipoma and liposarcoma [5]. Macroscopically, myelolipomas are usually single [27,32], but multiple lesions in the same organ [2,14,17,22,23] or multicentric myelolipoma [18] have been described in some species. 

The case presented herein showed multiple clinicopathologic abnormalities across serial testing. Initially, only a mild increase in serum ALT activity was noted. This could be due to systemic malaise (e.g., hyporexia, weight loss) causing non-specific hepatocyte necrosis, subclinical chronic hepatitis or hyperthyroidism. Hyperthyroidism was excluded in the case as the Total T4 was normal. On a second set of laboratory results, the ALT serum activity was still increased and slightly higher than in the previous assessment, which may not be clinically significant. Mild hyperglycaemia was most likely attributed to stress, whilst mildly increased CK activity and marginal hypotriglyceridaemia were thought to be insignificant in this case. Free hypercalcemia, a known cause of hyporexia in cats [33], was evident in our case on the initial screening. PTH and PTH-RP were both undetectable, ruling out hyperparathyroidism and making hypercalcemia of malignancy unlikely. There is no known association between myelolipoma and the development of hypercalcemia in mammals. Whilst idiopathic hypercalcemia of cats could not be completely ruled out in our case, the significance of this abnormality remains uncertain, especially as free calcium spontaneously normalised one week prior to surgery as demonstrated on subsequent two measurements. 

Post-operatively, the PCV dropped from the previous 32% to 30%. This change may be due to blood loss during surgery or haemodilution due to intravenous fluid therapy, and does not represent a significant reduction. Hyponatremia and hypokalaemia could be due to excess salivation, as the cat did show ptyalism following surgery. Hypochloraemia may be due to concurrent loss of sodium. This may have caused the ECG abnormalities in the post-operative period. The sudden onset of post-operative hypotension could have been due to post-operative haemorrhage, shock, acidosis, hypothermia, bradycardia or cardiac failure. The former was ruled out on the ultrasound scan, and underlying cardiac disease was unlikely due to a normal recent echocardiogram. The hypotension, hypothermia and electrolyte disturbances were managed medically, but may have predisposed to thrombus formation, which was speculated as a cause of death in this case, despite short-term improvement in clinical parameters. Serial coagulation profile testing was not available but could have been useful in substantiating the speculation that clotting abnormalities were implicated in the outcome of this patient. 

A small case series showed that splenectomy is well tolerated in the cat, but this study is retrospective and includes various diagnoses which may have an impact on survival [34]. One case of myelolipoma was included, which had a mean survival time of 339 days (alive at the time of publication) [34]. The report indicated pre-operative weight loss as a negative prognostic indicator for survival, which may be relevant to our case. 

In another study, 5 out of 36 cases (15%) that underwent splenectomy for treatment of a splenic mast cell tumour (MCT) died or were euthanised in the 72 h following surgery [35]. The causes of death included cardiopulmonary arrest and suspected degranulation episode. One cat was euthanised due to persistent haemorrhage and suspected disseminated intravascular coagulation (DIC). Reports show that splenectomy can positively impact mean survival times in cats with splenic mast cell tumours (MCTs) [35,36]. Splenectomy is consequently considered the treatment of choice for splenic MCTs in cats. However, MCTs are malignant tumours, whereas splenic myelolipomas are considered benign, so the survival times may not be comparable. 

There is a risk of death in up to 2% of sick cats undergoing general anaesthesia, and splenectomy also carries a risk of haemorrhage, vascular compromise, arrhythmias, systemic inflammatory response syndrome (SIRS), DIC, hypoxia and ischemia, all of which may be fatal [37,38]. Splenectomy has been associated with a rapid reactive thrombocytosis due to decreased cell degradation [39]. This, taken together with an enhanced generation of thrombin in splenectomised patients, can lead to an increased risk of subsequent thromboembolism [40], which was speculated as a cause of death in the case presented herein. Ultimately, the risks of surgery need to be balanced against the risk of hemoabdomen associated with the splenic neoplasm [2,15]. There is a scarcity of evidence to support medical versus surgical treatment options in feline patients for splenic myelolipoma due to a lack of long-term follow-up in patients diagnosed with this rare disease. 

## 4. Conclusions

In conclusion, splenic myelolipomas are infrequently encountered in feline practice. Whilst conservative management may be appropriate in asymptomatic cases, in patients where clinical signs are attributable to the perceived mass effect of myelolipoma, surgery, although not without risk, needs to be considered on a case-by-case basis, taking into consideration individual patient factors. 

## Data Availability

The original contributions presented in the study are included in the article, further inquiries can be directed to the corresponding author.
